# Environmental Enrichment Induces Meningeal Niche Remodeling through TrkB-Mediated Signaling

**DOI:** 10.3390/ijms221910657

**Published:** 2021-10-01

**Authors:** Stefania Zorzin, Andrea Corsi, Francesca Ciarpella, Emanuela Bottani, Sissi Dolci, Giorgio Malpeli, Annachiara Pino, Alessia Amenta, Guido Franceso Fumagalli, Cristiano Chiamulera, Francesco Bifari, Ilaria Decimo

**Affiliations:** 1 Section of Pharmacology, Department of Diagnostic and Public Health, University of Verona, 37134 Verona, Italy; stefania.zorzin@univr.it (S.Z.); andrea.corsi@univr.it (A.C.); Francesca.ciarpella@univr.it (F.C.); emanuela.bottani@univr.it (E.B.); sissi.dolci@univr.it (S.D.); annachiara.pino@gmail.com (A.P.); guido.fumagalli@univr.it (G.F.F.); cristiano.chiamulera@univr.it (C.C.); 2 Department of Surgical Sciences, Dentistry, Gynecology and Pediatrics, University of Verona, 37134 Verona, Italy; giorgio.malpeli@univr.it; 3 Laboratory of Cell Metabolism and Regenerative Medicine, Department of Medical Biotechnology and Translational Medicine, University of Milan, 20129 Milan, Italy; alessia.amenta@guest.unimi.it (A.A.); francesco.bifari@unimi.it (F.B.)

**Keywords:** neurogenesis, TrkB, BDNF, meninges, ANA-12, meningeal niche, radial glia cell, neural precursor, enriched environment, EE, ENR

## Abstract

Neural precursors (NPs) present in the hippocampus can be modulated by several neurogenic stimuli, including environmental enrichment (EE) acting through BDNF-TrkB signaling. We have recently identified NPs in meninges; however, the meningeal niche response to pro-neurogenic stimuli has never been investigated. To this aim, we analyzed the effects of EE exposure on NP distribution in mouse brain meninges. Following neurogenic stimuli, although we did not detect modification of the meningeal cell number and proliferation, we observed an increased number of neural precursors in the meninges. A lineage tracing experiment suggested that EE-induced β3-Tubulin^+^ immature neuronal cells present in the meninges originated, at least in part, from GLAST^+^ radial glia cells. To investigate the molecular mechanism responsible for meningeal reaction to EE exposure, we studied the BDNF-TrkB interaction. Treatment with ANA-12, a TrkB non-competitive inhibitor, abolished the EE-induced meningeal niche changes. Overall, these data showed, for the first time, that EE exposure induced meningeal niche remodeling through TrkB-mediated signaling. Fluoxetine treatment further confirmed the meningeal niche response, suggesting it may also respond to other pharmacological neurogenic stimuli. A better understanding of the neurogenic stimuli modulation for meninges may be useful to improve the effectiveness of neurodegenerative and neuropsychiatric treatments.

## 1. Introduction

Mammal adult neurogenesis can be modulated by several stimuli, including exposure to environmental enrichment (EE) [1,2] and antidepressant treatments [3,4,5,6].

EE has been shown to induce hippocampal neurogenesis, and its therapeutic effect has been used to ameliorate brain injury outcomes [7]. Similarly, fluoxetine (FLUOX), a selective serotonin reuptake inhibitor (SSRI) antidepressant [3,8], as well as other SSRI antidepressants like imipramine, also increase hippocampal neurogenesis [3,4,9]. The molecular mechanisms responsible for the induction of hippocampal neurogenesis modulation by neurogenic stimuli have not been fully elucidated. However, a major role of the BDNF-TrkB signaling pathway has been described by several groups [10,11,12,13]. Aside from the effects of neurogenic stimuli on hippocampal neurogenesis, little is known about their effects on other less-studied brain neural stem cell (NSC) niches [14].

Recently, a subset of meningeal-residing neural progenitors (NPs) has been identified during both development and adulthood in different mammals, including humans [14,15,16,17,18,19]. Single-cell RNA sequencing (scRNAseq) analysis of the meningeal cells identified a small fraction of cells with a signature corresponding to radial glia-like cells, expressing GLAST and a population with a neuroblast signature expressing β3-Tubulin [20]. These NPs can migrate from the meninges to the brain parenchyma and differentiate into functional cortical neurons or oligodendrocytes [20,21]. The meningeal stem cell niche is composed of specific trophic extracellular matrix components (ECMs) called fractones, and it is able to sense and respond to neurotrophic stimuli including FGF2, NGF and EGF [22,23,24]. It is noteworthy that the NPs present in meninges have been shown to react to CNS diseases, including spinal cord and brain injuries, stroke and progressive ataxia [16,25]. Meninges may therefore represent a functional niche for NPs. However, the response of NPs in meninges to neurogenic stimuli such as EE tasks [26] or drugs [27] (e.g., fluoxetine) has never been assessed. In this work, we investigate the effects and the molecular mechanisms of the meningeal niche response to EE exposure.

We found that EE exposure induced meningeal niche remodeling by increasing the number of NPs in the meninges. Lineage tracing experiments showed that the EE-induced NPs in meninges were derived from radial glia GLAST^+^ cells. Pharmacological inhibition of the TrkB receptor following EE exposure abolished both the hippocampal and meningeal increase in NPs, supporting the role of TrkB-BDNF signaling in the hippocampal and meningeal responses to EE. Fluoxetine administration further confirmed that the meningeal niche response was not restricted to EE exposure, and it may be extended to other neurogenic stimuli.

## 2. Results

### 2.1. EE Exposure Induces Meningeal Niche Remodeling

**Figure 1:**
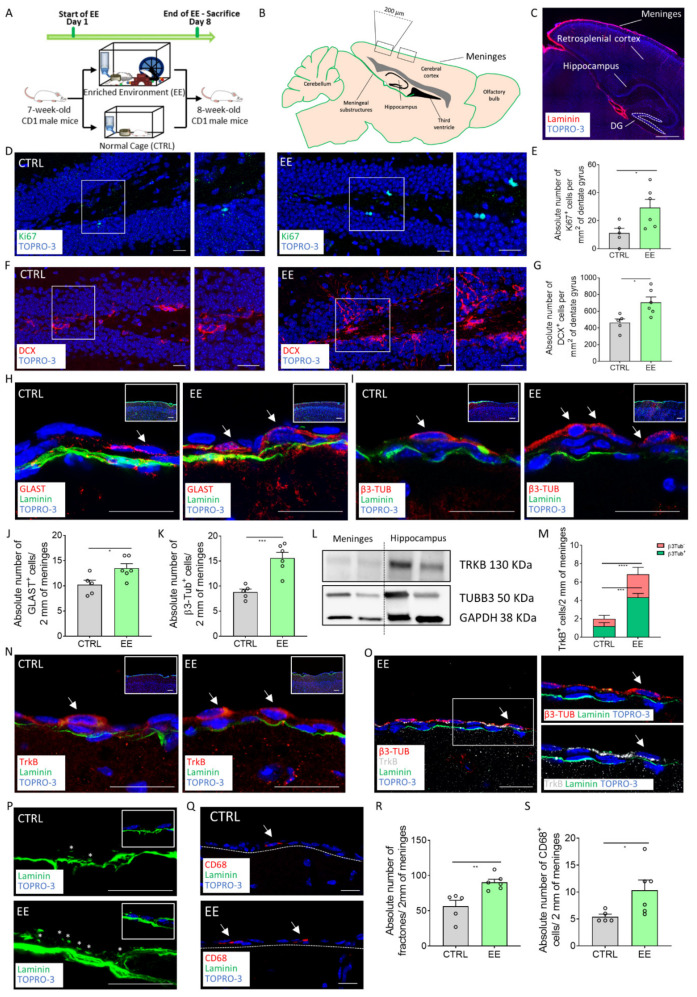
The meningeal niche is responsive to enriched environment exposure. (**A**) Schematic representation of the experimental design used for EE exposure. (**B**) Scheme depicting the sagittal section of the mouse brain, highlighting the specific regions where brain external meninges were analyzed. (**C**) Sagittal brain section of CD1 mice with laminin (red) identifying the brain meninges. White dashed lines delineate the dentate gyrus (DG) area used for quantification. (**D**) Sagittal brain section of CD1 mice showing hippocampal Ki67^+^ (green) cells in non-treated (CTRL) and treated (EE) animals. A white box highlights a DG zone, reported as a magnification on the right panel. (**E**) Graph showing the number of Ki67^+^ cells per mm^2^ of the DG in the CTRL and EE animals. (**F**) Sagittal brain sections of CD1 mice showing hippocampal DCX^+^ (red) cells in the CTRL and EE mice. A white box highlights a DG zone reported as a magnification on the right panel. (**G**) Graph showing the number of DCX^+^ cells per mm^2^ of the DG in the CTRL and EE animals. (**H**) Sagittal brain sections of CD1 mice showing GLAST^+^ (red) cells above the meningeal basal lamina (green) in the CTRL and EE mice. An insert illustrates a panoramic image where the single-cell level image was taken. (**I**) Sagittal brain sections of CD1 mice showing β3-Tubulin^+^ (red) cells above the meningeal basal lamina (green) in the CTRL and EE mice. An insert illustrates a panoramic image where the single-cell level image was taken. (**J**) Graph showing the number of GLAST^+^ cells in 2 mm of the retrosplenial brain meninges of the CTRL and EE animals. (**K**) Graph showing the number of β3-Tubulin^+^ cells in 2 mm of the retrosplenial brain meninges of the CTRL and EE animals. (**L**) Representative western blot analysis of mouse brain protein extracts, which shows the presence of TrkB and β3-Tubulin proteins in both the mouse meningeal samples and the hippocampal samples. (**M**) Graph showing TrkB^+^/β3-Tubulin^+^ and Trkb^+^/β3-Tubunlin^−^ cells in 2 mm of the retrosplenial brain meninges of the CTRL and EE animals. (**N**) Sagittal brain sections of CD1 mice showing TrkB^+^ (red) cells above the meningeal basal lamina (green) in the CTRL and EE mice. An insert illustrates a panoramic image where the single-cell level image was taken. (**O**) Sagittal brain section of CD1 mice exposed to an EE showing a cell double positive for β3-Tubulin (red) and TrkB (white) above the meningeal basal lamina (green). A white box highlights a double positive cell, reported as a magnification on the right panel. (**P**) Sagittal brain sections of CD1 mice showing fractones (*) identified via laminin (green) staining in the retrosplenial brain meninges of the CTRL and EE mice. An insert illustrates laminin staining (fractones) below the meningeal nuclei (TOPRO-3). (**Q**) Sagittal brain sections of CD1 mice showing CD68^+^ (red) cells in the brain meninges of the CTRL and EE mice. The meninges are delineated using white dashes. (**R**) Graph showing the number of fractones in 2 mm of the retrosplenial brain meninges of the CTRL and EE animals. (**S**) Graph showing the number of CD68^+^ cells in 2 mm of the retrosplenial brain meninges of the CTRL and EE animals. Data are presented as the mean ± SEM. * *p* value ≤ 0.05. ** *p* value ≤ 0.01. *** *p* value ≤ 0.001. **** *p* value ≤ 0.0001. In (**C**,**D**,**F**,**H**,**I**,**N**–**Q**), the nuclei are in blue (TOPRO-3 nuclear staining). (**C**,**O**,**Q**) are single-plane confocal images. (**D**,**F**,**H**,**I**,**N**,**P**) are the maximum intensity projections of the z-stack confocal images. The scale bars represent 500 µm (**C**), 20 µm in the magnified pictures (**D**,**F**,**H**,**I**,**N**,**O**,**P**,**Q**) and 100 µm in the panoramic anchor images (**H**,**I**,**N**). White arrows indicate positive cells, while asterisks indicate fractones.

EE is a pro-neurogenic stimulus known to induce hippocampal neurogenesis. In order to assess the effectiveness of the EE treatment on the meningeal niche, we exposed to an EE CD1 mice for 7 days and then analyzed the sagittal mouse brain sections at the retrosplenial cortical level by immunofluorescence and confocal analysis (Figure 1A–C). To validate the EE treatment, we first assessed its known impact on hippocampal neurogenesis [10,28,29] by analyzing the number of cells expressing the proliferation marker Ki67 or the immature neuronal marker DCX in the dentate gyrus (DG) (Figure 1D–G). The number of Ki67^+^ and DCX^+^ cells in the DGs of the treated mice was significantly higher compared with the control mice (number of Ki67^+^ cells per mm^2^ of DG area: CTRL = 11.06 ± 3.513, *n* = 5; EE = 29.31 ± 5.876, *n* = 6; *p* = 0.0325; number of DCX^+^ cells per mm^2^ of DG: CTRL = 463 ± 44.57, *n* = 5; EE = 706.5 ± 62.84, *n* = 6; *p* = 0.0142) (Figure 1E,G). In line with the literature data, these results confirmed that the EE was effective and it increased hippocampal neurogenesis [10,28,29]. To investigate if the EE affected the meningeal niche, we first analyzed the number of cell nuclei and the expression of Ki67 in 2 mm (5 brain sections, 2 segments of 200 µm in each section for each animal) of cross-sectioned brain retrosplenial meninges (Figure 1B). We did not observe differences in either the total cell number or the proliferation index in the meninges of the control or EE-treated groups (Figure S1).

The NPs present in retrosplenial meninges are characterized by features of radial glia-like cells expressing GLAST and immature neurons expressing β3-Tubulin [16]. Therefore, we analyzed in the retrosplenial meninges the distribution of radial glia-like cells expressing GLAST and immature neurons expressing β3-Tubulin (Figure 1H–K). We found a significant increase in the GLAST^+^ cells and β3-Tubulin^+^ cells in the treated mice compared with the control group (number of GLAST^+^ cells per 2 mm of cross-sectioned meninges: CTRL = 10.2 ± 0.9028, *n* = 5; EE = 13.5 ± 0.8756, *n* = 6; *p* = 0.0284; number of β3-Tubulin^+^ cells per 2 mm of cross-sectioned meninges: CTRL = 8.8 ± 0.6042, *n* = 5; EE = 15.58 ± 1.158, *n* = 6; *p* = 0.0009) (Figure 1J,K). These data indicate that the meningeal niche responded to the EE neurogenic stimulus by increasing the NP cell number.

It is known that EE-mediated neurogenesis is carried out via molecular mediators, including the BDNF-TrkB signaling pathway [3,10]. TrkB was identified as a gene expressed by the meningeal niche [20]. To investigate if this pathway was involved in the meningeal response to neurogenic stimuli, we first confirmed the expression of TrkB and β3-Tubulin in the meningeal cells via western blot analysis (Figure 1L). We then proceeded to analyze the retrosplenial meninges through immunofluorescence and confocal analysis of mice exposed to the EE. We found a significant increase in the TrkB^+^ retrosplenial meningeal cell population in response to EE exposure (number of TrkB^+^ cells per 2 mm of cross-sectioned meninges: CTRL = 2 ± 0.5477, *n* = 5; EE = 6.833 ± 0.4773, *n* = 6; *p* < 0.0001) (Figure 1M–O). In addition, we identified a rare population of immature neurons co-expressing β3-Tubulin and the BDNF receptor TrkB. While this double positive population was extremely sporadic in the control animals, it was significantly increased in the EE-exposed mice (number of β3-Tubulin^+^/^+^TrkB^+^ cells per 2 mm of cross-sectioned meninges: CTRL = 1.2 ± 0.3742, *n* = 5; EE = 4.333 ± 0.4216, *n* = 6; *p* = 0.0004) treatment groups (Figure 1M,O).

The meningeal niche is endowed with fractones, small laminin-based structures able to capture growth factors and exert a trophic role for the neural stem cell niche [30], and immune cells fundamental to maintaining neurogenesis [31]. We therefore evaluated the distribution of the fractones in the meninges by analyzing the laminin^+^ puncta [15] and the number the macrophages identified by the CD68 marker (Figure 1P–S). We found a statistically significant increase in the meningeal fractones and in the CD68^+^ cells after exposure to EE (number of fractones per 2 mm of cross-sectioned meninges: CTRL = 56.2 ± 8.599, *n* = 5; EE = 90.25 ± 4.393, *n* = 6; *p* = 0.0048; number of CD68^+^ cells per 2 mm of cross-sectioned meninges: CTRL = 5.432 ± 0.4638, *n* = 5; EE = 10.34 ± 1.878, *n* = 6; *p* = 0.0456; Figure 1R,S). These data further indicated that the EE induced changes in the meningeal ECM and macrophages, suggesting a trophic activation of the meningeal niche.

Overall, these results showed that EE exposure induced meningeal niche remodeling by modulation of the NPs, immature neurons, fractones and macrophages, supporting the responsiveness of the meningeal niche to pro-neurogenic stimuli.

### 2.2. EE-Induced NPs in Meninges Derived from GLAST^+^ Radial Glia Progenitors

**Figure 2:**
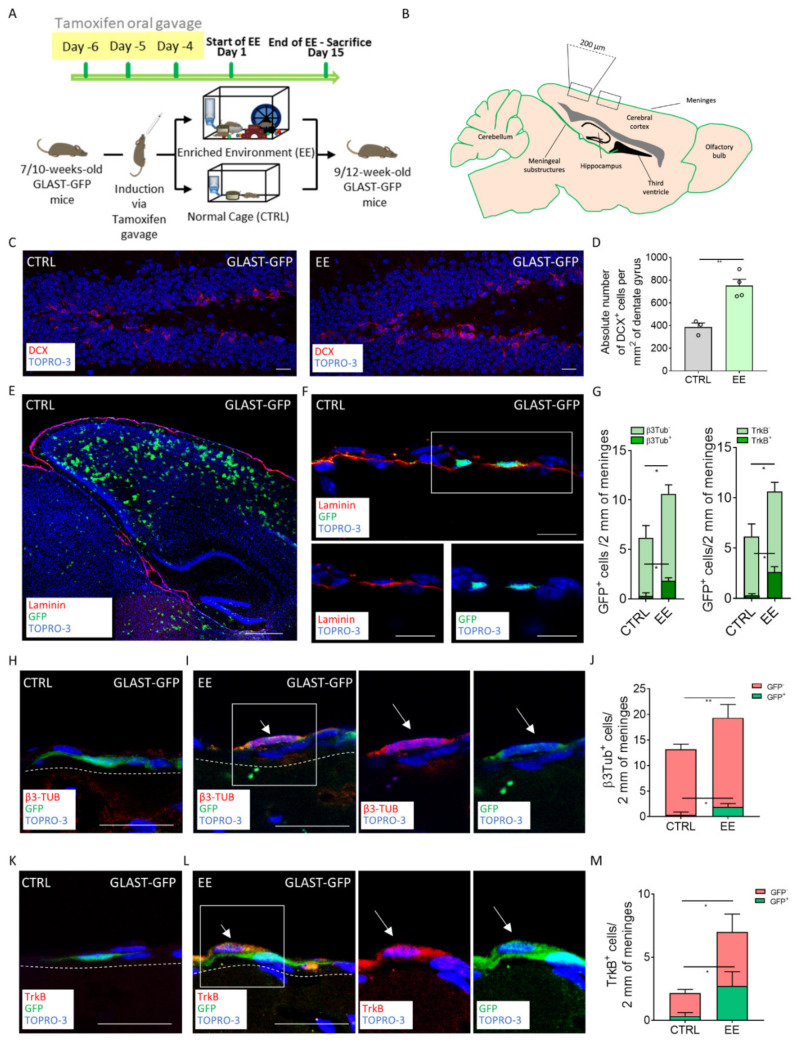
Lineage tracing confirms the radial glial origin of the EE-induced immature neurons. (**A**) Schematic representation of the experimental design used for EE exposure for the GLAST-GFP mice. (**B**) Scheme depicting the sagittal section of a mouse brain, highlighting the specific regions where the brain external meninges were analyzed. (**C**) Sagittal brain section of GLAST-GFP mice, showing hippocampal DCX^+^ (red) cells in the CTRL and EE mice. (**D**) Graph showing the number of DCX^+^ cells per mm^2^ of the dentate gyri (DG) in the CTRL and EE animals. (**E**) Sagittal brain section of the GLAST-GFP CTRL mice, with laminin (red) identifying the brain meninges and GFP (green) identifying the transgene expression. (**F**) Sagittal brain section of the GLAST-GFP mice, showing GFP^+^ (green) cells above the meningeal basal lamina identified via laminin (red) staining. A white box highlights the GFP-positive cells, reported as split channels on the lower panel. (**G**) Graphs showing GFP^+^/β3-Tubulin^+^ and GFP^+^/β3-Tubulin^−^ cells (left) and GFP^+^/TrkB^+^ and GFP^+^/TrkB^−^ cells (right). (**H**) Sagittal brain section of the GLAST-GFP CTRL mice, showing a cell only positive for GFP (green). White dashes delineate the meninges. (**I**) Sagittal brain section of the GLAST-GFP mice, showing a cell positive for both β3-Tubulin (red) and GFP (green) in the EE mice. A white box highlights a double positive cell reported as split channels on the right. White dashes delineate the meninges. (**J**) Graph showing the β3-Tubulin^+^/GFP^+^ and β3-Tubulin^+^/GFP^−^ cells in 2 mm of the retrosplenial brain meninges of the CTRL and EE animals. (**K**) Sagittal brain section of the GLAST-GFP CTRL mice, showing a cell only positive for GFP (green). White dashes delineate the meninges. (**L**) Sagittal brain section of the GLAST-GFP mice, showing a cell positive for both TrkB (red) and GFP (green) in the EE mice. A white box highlights a double positive cell reported as split channels on the right. White dashes delineate the meninges. (**M**) Graph showing the TrkB^+^/GFP^+^ and TrkB^+^/GFP^−^ cells in 2 mm of the retrosplenial brain meninges of the CTRL and EE animals. Data are presented as the mean ± SEM. * *p* value ≤ 0.05. ** *p* value ≤ 0.01. In (**C**,**E**,**F**,**H**,**I**,**K**,**L**), the nuclei are in blue (TOPRO-3 nuclear staining). (**E**,**F**) are single-plane confocal images. (**C**,**H**,**I**,**K**,**L**) are maximum-intensity projections of z-stack confocal images. The scale bars represent 20 µm in pictures (**C**,**F**,**H**,**I**,**K**,**L**) and 500 µm in (**E**). White arrows indicate positive cells.

To identify the lineage of the immature neurons, which increased in the meninges after the exposure to EE, we took advantage of an inducible transgenic mouse model for the radial glial cells. The GLAST-Cre^ERT2^ mice [32], intercrossed with the CAG-CAT-EGFP reporter line [33] (GLAST-GFP), allowed all the GLAST^+^ cells and their progeny to be labeled by GFP following tamoxifen administration. After 3 days of tamoxifen induction via oral gavage, we subjected the mice to 15 days of the EE, and then we analyzed the brain and the retrosplenial meninges of the control and treated mice (Figure 2A,B) [32].

At first, we confirmed the effectiveness of the EE protocol in the GLAST-GFP mice by assessing the increased number of DCX cells in the DGs of the treated mice (Figure 2C,D) (number of DCX^+^ per mm^2^ of DG area, CTRL = 386.4 ± 36.7, *n* = 3:, *n* = 4; EE = 751.8 ± 56.24, *n* = 4; *p* = 0.0042). The time of the exposure to the EE was increased to 15 days, as we could not observe the hippocampal neurogenic response in the GLAST-GFP mice after 7 days, possibly due to gavage administration of tamoxifen (data not shown) [34,35,36].

We then analyzed the distribution and fate of the GFP^+^ GLAST-derived progenitors. As expected, we found GFP^+^ cells in the cortex, DG, striatum and meninges both in the EE-treated and the control mice (Figure 2E,F) [32]. We observed a statistical increase in the GFP^+^ cells in the meninges after exposure to EE (number of GFP^+^ cells per 2 mm of cross-sectioned meninges: CTRL = 6.167 ± 1.244, *n* = 3; EE = 10.62 ± 0.9157, *n* = 4; *p* = 0.0315) (Figure 2G). Since tamoxifen-induced recombination was done before the EE exposure, and there was no change in the cell number of retrosplenial cortical meninges, the total increase in GFP^+^ cells may have arisen from migrating progenitors coming from other brain regions.

In order to assess the fate of the GFP^+^ cells in the retrosplenial meninges following EE exposure, we analyzed their co-expression of β3-Tubulin. We found the presence of a double positive GFP^+^/β3-Tubulin^+^ cell population in the EE-treated mice meninges (Figure 2H–J). On the contrary, double positive cells were almost absent in the control mice, potentially suggesting that some immature neurons differentiated from the GLAST-derived cells following EE exposure (number of GFP^+^/β3-Tubulin^+^ cells per 2 mm of cross-sectioned meninges: CTRL = 0.3333 ± 0.3333, *n* = 3; EE = 1.893 ± 0.3362, *n* = 4; *p* = 0.0237; Figure 2G,J). In line with the results obtained in the CD1 mice, following EE exposure, we observed also in C57Bl6 mice an overall increase in β3-Tubulin^+^ cells in the meninges (Figure 2J) (number of β3-Tubulin^+^ cells per 2 mm of cross-sectioned meninges: CTRL = 13.17 ± 0.6009, *n* = 3; EE = 19.33 ± 1.113, *n* = 4; *p* = 0,0072).

We further confirmed the increased expression of TrkB/GFP^+^ cells in the meninges (Figure 2K–L) (number of GFP^+^/TrkB^+^ cells per 2 mm of cross-sectioned meninges: CTRL = 0.3333 ± 0.1667, *n* = 3; EE = 2.72 ± 0.5725, *n* = 4; *p* = 0.0182; number of TrkB^+^ cells per 2 mm of cross-sectioned meninges: CTRL = 2.167 ± 0.1667, *n* = 3; EE = 7 ± 1.186, *n* = 4; *p* = 0.0187) (Figure 2G,M), suggesting that they were partially derived from the GLAST^+^ radial glia lineage.

Altogether, these data indicated that following EE exposure, the increased β3-Tubulin^+^ immature neurons and TrkB^+^ cells in the meninges partially originated from the radial glia GLAST^+^ cells.

### 2.3. TrkB/BDNF Signaling Modulates the Meningeal Niche Response to Enriched Environment Exposure

**Figure 3:**
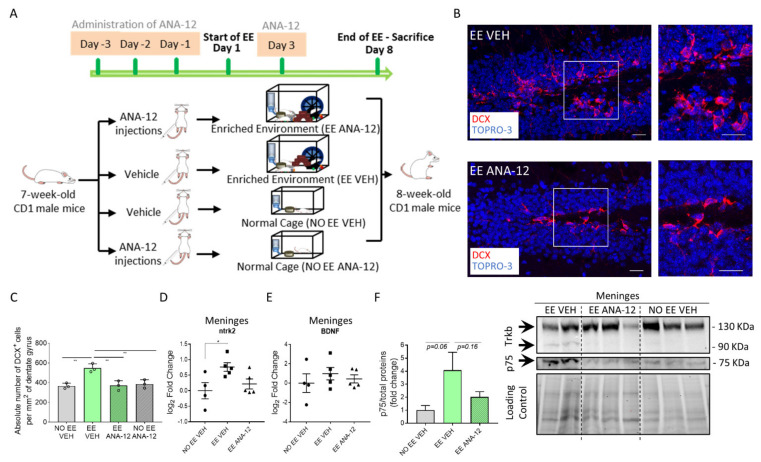
ANA-12 inhibits hippocampal neurogenesis and impacts BDNF-TrkB signaling in the meninges. (**A**) Schematic representation of the experimental design used for EE exposure and TrkB inhibitor ANA-12 administration in CD1 mice. (**B**) Sagittal brain sections of CD1 mice, showing hippocampal DCX^+^ (red) cells in animals exposed to the EE and administered only the vehicle (EE VEH) and animals exposed to the EE and administered with the TrkB inhibitor ANA-12 (EE ANA-12). A white box highlights a dentate gyrus (DG) zone, reported as a magnification on the right panel. (**C**) Graph showing the number of DCX^+^ cells per mm^2^ of the DG in animals subjected to the EE or not and administered with the vehicle or ANA-12 (NO EE VEH, EE VEH, EE ANA-12 and NO EE ANA-12). (**D**) Graph depicting gene expression analysis for *ntrk2* (TrkB) in NO EE VEH, EE VEH and EE ANA-12 samples. Expression levels are reported as the log_2_ fold change with respect to NO EE VEH. (**E**) Graph depicting gene expression analysis for BDNF in NO EE VEH, EE VEH and EE ANA-12 mice. Expression levels are reported as the log_2_ fold change with respect to the NO EE VEH mice. (**F**) Graph showing the differential fold change in protein expression for the p75 protein in NO EE VEH, EE VEH and EE ANA-12 mice with respect to the NO EE VEH samples (left panel). A representative western blot analysis of the mouse brain meningeal protein extracts shows the presence of TrkB and p75 proteins in the EE VEH, EE ANA-12 and NO EE VEH samples (right). In the EE VEH samples, one additional TrkB protein isoform was identified. Data are presented as the mean ± SEM. * *p* value ≤ 0.05. ** *p* value ≤ 0.01. In (**B**), the nuclei are in blue (TOPRO-3 nuclear staining). (**B**) shows the maximum intensity projections of the z-stack confocal images. The scale bars represent 20 µm.

We found that TrkB was expressed in the meninges, and following EE exposure, it increased in association with the β3-Tubulin meningeal progenitors (Figure 1M–O). TrkB receptor activation is suppressed by ANA-12 [37], a non-competitive inhibitor. In order to provide evidence that the meningeal response was directly regulated by the activation of the neurotrophic receptor TrkB, we administrated ANA-12 to CD1 mice 3 days before and at the third day of EE exposure (Figure 3A) [38]. In order to compare the effect of EE exposure and EE exposure plus the ANA-12 inhibitor, we set up the following experimental groups: EE ANA-12 (animals exposed to EE and injected with the inhibitor), EE VEH (animals exposed to EE and injected with the vehicle), NO EE VEH (single-housed animals receiving just vehicle injections) and NO EE ANA-12 (single-housed animals receiving the inhibitor) (Figure 3A).

At first, we assessed if the inhibitor was able to ablate the canonical effects caused by the EE on the hippocampal neurogenesis. As expected, ANA-12 ablated the EE neurogenic effects on the DG (number of DCX^+^ cells per 2 mm of DG area: NO EE VEH = 366.1 ± 16.16, *n* = 3; EE VEH = 547.1 ± 25.39, *n* = 3; EE ANA-12: 370.7 ± 27.76, *n* = 3; NO EE ANA-12: 386.6 ± 24.68, *n* = 3; NO EE VEH vs. EE VEH, *p*= 0.0030; EE VEH vs. EE ANA-12, *p* = 0.0036; EE VEH vs. NO EE ANA-12, *p* = 0.0063) (Figure 3B,C). These results validated the effect of ANA-12 on the hippocampal neurogenesis.

We then verified whether ANA-12 affected the expression of TrkB/BDNF in the meninges. We found that *ntrk2* (the gene name for TrkB) significantly increased its gene expression in meninges following EE exposure and decreased when the EE exposure was associated with ANA-12 administration (log_2_ fold change: NO EE VEH = 0 ± 0.26, *n* = 4; EE VEH = 0.76 ± 0.14, *n* = 5; EE ANA-12 = 0.22 ± 0.16, *n* = 5; NO EE VEH vs. EE VEH, *p* = 0.0272) (Figure 3D). qRT-PCR indicated BDNF expression in the meninges which did not change following EE exposure or EE plus ANA-12 administration (log_2_ fold change: NO EE VEH = 0 ± 0.96, *n* = 4; EE VEH = 0.98 ± 0.64, *n* = 5; EE ANA-12 = 0.44 ± 0.42, *n* = 5) (Figure 3E). Western blot analysis showed that, following EE exposure, TrkB increased the expression of the truncated 1 isoform (TrkB.T1: 90 KDa) [39], which decreased to an undetectable control level following ANA-12 administration (Figure 3F). The full-length TrkB (130 KDa) did not change its expression among the different groups. To further confirm the increase in the expression of the TrkB signaling pathway in the meninges following EE exposure, we assessed the expression of the neurotrophin receptor p75 [40,41]. The p75 receptor cross links with the TrkB receptor to activate the response to BDNF signaling. Indeed, we found that p75 increased following EE exposure and decreased after ANA-12 administration (p75/total proteins (fold change): NO EE VEH = 1 ± 0.36, *n* = 3; EE VEH = 4.08 ± 1.38, *n* = 2; EE ANA-12 = 2.03 ± 0.4, *n* = 3; NO EE VEH vs. EE VEH, *p* = 0.0695) (Figure 3F).

We then assessed if the changes observed in the meninges after EE exposure could be reversed by blocking the TrkB/BDNF signaling.

**Figure 4:**
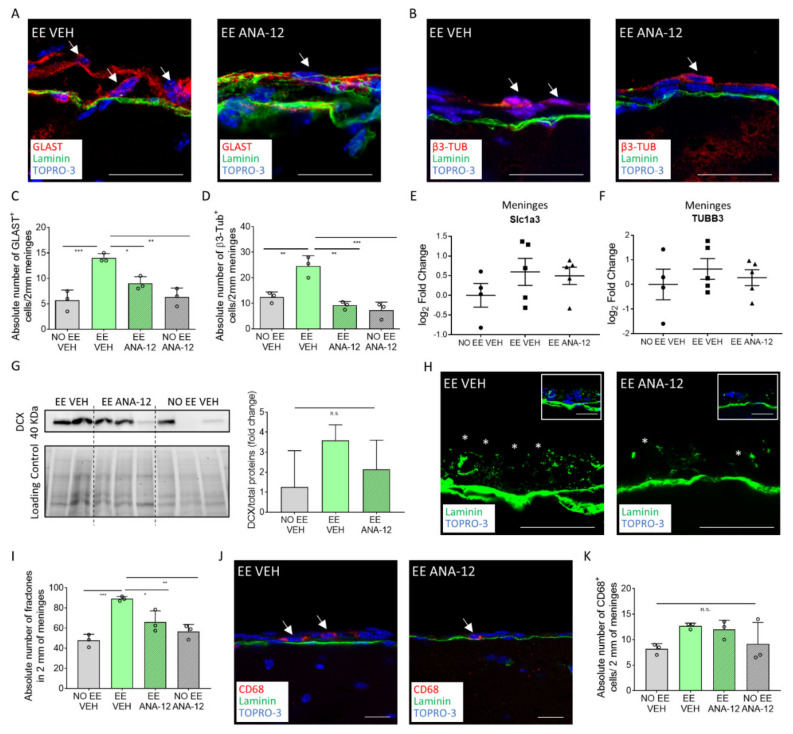
ANA-12 administration reverts EE-induced changes in the meningeal niche. (**A**) Sagittal brain sections of the CD1 mice, showing GLAST^+^ (red) cells above the meningeal basal lamina (green) in the EE VEH and EE ANA-12 mice. (**B**) Sagittal brain sections of the CD1 mice, showing β3-Tubulin^+^ (red) cells above the meningeal basal lamina (green) in the EE VEH and EE ANA-12 mice. (**C**) Graph showing the number of GLAST^+^ cells in 2 mm of the retrosplenial brain meninges of the NO EE VEH, EE VEH, EE ANA-12 and NO EE ANA-12 animals. (**D**) Graph showing the number of β3-Tubulin^+^ cells in 2 mm of the retrosplenial brain meninges of the NO EE VEH, EE VEH, EE ANA-12 and NO EE ANA-12 animals. (**E**) Graph depicting gene expression analysis for *Slc1a3* (GLAST) in the NO EE VEH, EE VEH and EE ANA-12 mice. Expression levels are reported as the log_2_ fold change with respect to the NO EE VEH mice. (**F**) Graph depicting gene expression analysis for *TUBB3* (β3-Tubulin) in the NO EE VEH, EE VEH and EE ANA-12 mice. Expression levels are reported as the log_2_ fold change with respect to the NO EE VEH mice. (**G**) Representative western blot analysis of mouse brain meningeal protein extracts, which shows the presence of the DCX protein in the EE VEH, EE ANA-12 and NO EE VEH samples (left panel). The graph shows the differential fold change in protein expression for the DCX protein in the NO EE VEH, EE VEH and EE ANA-12 mice with respect to the NO EE VEH mice (right panel). (**H**) Sagittal brain sections of the CD1 mice, showing fractones (*) identified via laminin (green) staining in the brain meninges of EE VEH and EE ANA-12 mice. An insert illustrates laminin staining (fractones) below the meningeal nuclei (TOPRO-3). (**I**) Graph showing the number of CD68^+^ cells in 2 mm of the retrosplenial brain meninges of the NO EE VEH, EE VEH, EE ANA-12 and NO EE ANA-12 animals. (**J**) Sagittal brain sections of the CD1 mice showing CD68^+^ (red) cells in the brain meninges of the EE VEH and EE ANA-12 mice. (**K**) Graph showing the number of fractones in 2 mm of the retrosplenial brain meninges of the NO EE VEH, EE VEH, EE ANA-12 and NO EE ANA-12 animals. Data are presented as the mean ± SEM, and n.s. = not statistically significant. * *p* value ≤ 0.05. ** *p* value ≤ 0.01. *** *p* value ≤ 0.001. In (**A**,**B**,**H**,**J**), the nuclei are in blue (TOPRO-3 nuclear staining). (**J**) is a single-plane confocal images. (**A**,**B**,**H**) are the maximum intensity projections of the z-stack confocal images. The scale bars represent 20 µm. White arrows indicate positive cells, while asterisks indicate fractones.

We found that the GLAST^+^ progenitors decreased in the EE-treated group after the co-administration of ANA-12 (number of GLAST^+^ cells per 2 mm of cross-sectioned meninges: NO EE VEH = 5.667 ± 1.167, *n* = 3; EE VEH = 14 ± 0.5, *n* = 3; EE ANA-12 = 9 ± 0.7638, *n* = 3; NO EE ANA-12 = 6.333 ± 1.014, *n* = 3; NO EE VEH vs. EE VEH, *p* = 0.0008; EE VEH vs. EE ANA-12, *p* = 0.0181; EE VEH vs. NO EE ANA-12, *p* = 0.0014) (Figure 4A,C). We also observed that the EE-induced increase in the β3-Tubulin^+^ population was completely ablated when the TrkB inhibitor was administered (Figure 4B,D) (number of β3-Tubulin^+^ cells per 2 mm of cross-sectioned meninges: NO EE VEH = 12.33 ± 1.202, *n* = 3; EE VEH = 24.5 ± 2.363, *n* = 3; EE ANA-12 = 9.167 ± 0.8819, *n* = 3; NO EE ANA-12 = 7.22 ± 1.848, *n* = 3; NO EE VEH vs. EE VEH, *p* = 0.0053; EE VEH vs. EE ANA-12, *p* = 0.0012; EE VEH vs. NO EE ANA-12, *p* = 0.0005). Gene expression analysis of the meningeal cells of the entire brain further confirmed the expression of *Slc1a3* (GLAST) and *TUBB3* (β3-Tubulin) genes in the meninges (*Slc1a3* log_2_ fold change: NO EE VEH = 0 ± 0.23, *n* = 4; EE VEH = 0.6 ± 0.34, *n* = 5; EE ANA-12 = 0.5 ± 0.22, *n* = 5; *TUBB3* log_2_ fold change: NO EE VEH = 0 ± 0.64, *n* = 4; EE VEH = 0.63 ± 0.42, *n* = 5; EE ANA-12 = 0.28 ± 0.33, *n* = 5) (Figure 4E,F).

To further confirm the TrkB signaling modulation of the meningeal neural progenitors following EE exposure, we assessed the expression of DCX, a different neural progenitor marker. DCX-positive cells in meninges are extremely rare in adult mice [15], however we were able to observe an increase in DCX protein in meninges following EE exposure. This increment was partially reduced in the EE plus ANA12 mice (DCX/total proteins (fold change): NO EE VEH = 1.25 ± 0.74, *n* = 3; EE VEH = 3.58 ± 0.39, *n* = 2; EE ANA-12 = 2.14 ± 0.6, *n* = 3) (Figure 4G). 

These data suggest that the administration of ANA-12 may interfere with the potential of meningeal β3-Tubulin^+^ immature neurons to react to EE.

We then assessed the effect of ANA-12 inhibition on the trophic and immune states of the meningeal niche. Interestingly, the increase in fractones in the meninges after EE exposure was reduced by ANA-12 administration, suggesting its regulation by TrkB/BDNF signaling (number of fractones per 2 mm of cross-sectioned meninges: NO EE VEH = 47.67 ± 3.632, *n* = 3; EE VEH = 89.17 ± 1.302, *n* = 3; EE ANA-12 = 66.17 ± 6.333, *n* = 3; NO EE ANA-12 = 56.5 ± 4.093, *n* = 3; NO EE VEH vs. EE VEH, *p* = 0.0006; EE VEH vs. EE ANA-12, *p* = 0.0207; EE VEH vs. NO EE ANA-12, *p* = 0.0027) (Figure 4H–I). We did not identify any reduction trend in the number of CD68^+^ cells in the meninges (Figure 4J–K).

Overall, the data suggested that the meningeal niche response to neurogenic EE stimulus was mediated by the neurotrophic receptor TrkB.

### 2.4. Fluoxetine Administration Induces the Meningeal Niche Response

Hippocampal adult neurogenesis can be enhanced by external stimuli such as antidepressant treatment [4,5,6]. Fluoxetine is an SSRI antidepressant which has been shown to ameliorate anxiety- or depression-related behavior and hippocampal neurogenesis [4].

**Figure 5:**
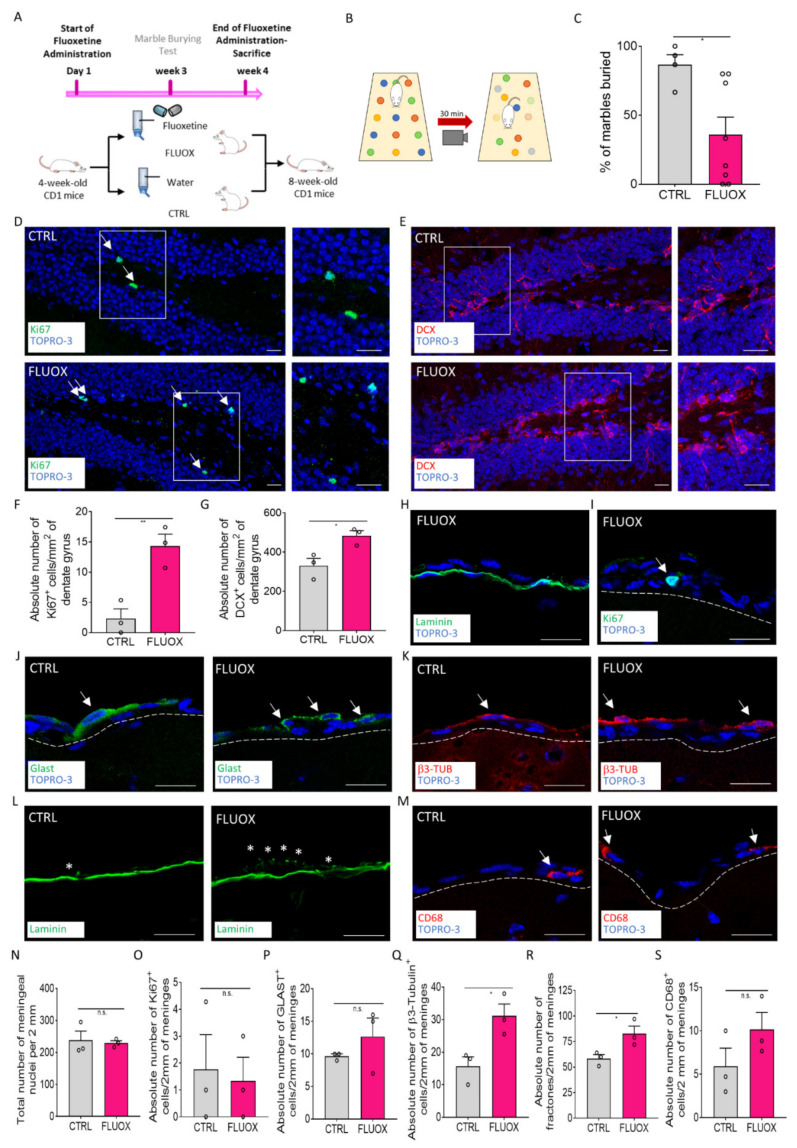
The meningeal niche responds to fluoxetine treatment. (**A**) Schematic representation of the experimental design used for fluoxetine administration. (**B**) Schematic representation of behavioral testing for OCD-like behavior via the marble burying test (MBT). (**C**) Graph showing the percentage of marbles buried by treated (FLUOX) and non-treated (CTRL) animals. (**D**,**E**) Sagittal brain sections of the CD1 mice, showing the presence of Ki67^+^ cells (green) and DCX^+^ cells (red) in the hippocampal dentate gyri (DGs) in the CTRL and FLUOX animals. A white box highlights a DG zone reported as a magnification on the right panel. (**F**) Graph showing the number of Ki67^+^ cells per mm^2^ of DG in the CTRL and FLUOX animals. (**G**) Graph showing the number of DCX^+^ cells per mm^2^ of DG in the CTRL and FLUOX animals. (**H**) Sagittal brain section of the CD1 mice, showing the brain meningeal nuclei and meningeal basal laminin (green) in a FLUOX animal. (**I**) Sagittal brain section of CD1 mice, showing a brain meningeal Ki67^+^ (green) cell in a FLUOX animal. The meninges are delineated via white dashes. (**J**) Sagittal brain sections of CD1 mice, showing brain meningeal GLAST^+^ cells in the CTRL and FLUOX mice. The meninges are delineated via white dashes. (**K**) Sagittal brain sections of CD1 mice, showing brain meningeal β3-Tubulin^+^ (red) cells in the CTRL and FLUOX mice. The meninges are delineated via white dashes. (**L**) Sagittal brain sections of CD1 mice, showing fractones (*) identified via laminin (green) staining in the brain meninges of the CTRL and FLUOX mice. (**M**) Sagittal brain sections of CD1 mice, showing brain meningeal CD68^+^ (red) cells in the CTRL and FLUOX mice. The meninges are delineated via white dashes. (N–S) Graphs showing the number of (**N**) nuclei, (**O**) Ki67^+^ cells, (**P**) GLAST^+^ cells, (**Q**) β3-Tubulin^+^ cells, (**R**) fractones and (**S**) CD68^+^ cells in 2 mm of the retrosplenial brain meninges of the CTRL and FLUOX animals. Data are presented as the mean ± SEM, and n.s. = not statistically significant. * *p* value ≤ 0.05. ** *p* value ≤ 0.01. In (**D**,**E**,**H**–**K**,**M**), the nuclei are in blue (TOPRO-3 nuclear staining). (**H**–**M**) are single-plane confocal images. (**D**,**E**) are maximum-intensity projections of z-stack confocal images. The scale bars represent 20 µm. White arrows indicate positive cells, while asterisks indicate fractones.

To investigate if a pro-neurogenic stimulus different from EE was able to induce changes in the meningeal niche, we exposed the CD1 mice (4 weeks old) to chronic (4 weeks) administration of fluoxetine as described by others (Figure 5A) [3,4,42].

We first confirmed the effectiveness of the fluoxetine treatment on animal behavior using an anxiety- and obsessive-compulsive disorder (OCD)-evaluating behavioral test (the marble burying test (MBT)) [43,44]. As shown in Figure 5B,C, following 4 weeks of drug administration, the percentage of marbles buried by the treated animals was significantly lower when compared with the controls (percentage of marbles buried over the total marble number: CTRL = 86.67% ± 7.2, *n* = 4; FLUOX = 35.83% ± 12.84, *n* = 8; *p* = 0.0246). As expected, the antidepressant was able to reduce OCD-like behavior and, thus, it suggested that fluoxetine exerted its pharmacological effect. We further confirmed fluoxetine’s efficacy on hippocampal neurogenesis by analyzing the expression of the proliferation marker Ki67 and DCX in the DG (Figure 5D–G). In line with previous observations, we found that the number of Ki67^+^ and DCX^+^ cells of the DGs were significantly higher in the fluoxetine-treated mice compared with the control mice (number of Ki67^+^ cells per mm^2^ of DG area: CTRL = 2.322 ± 1.592, *n* = 3; FLUOX = 14.33 ± 1.963, *n* = 3; *p* = 0.0090) (number of DCX^+^ cells per mm^2^ of DG area: CTRL = 331.3 ± 37.04, *n* = 3; FLUOX = 484.1 ± 25.58, *n* = 3; *p* = 0.0274) (Figure 5F,G). These data confirmed the fluoxetine treatment’s effectiveness, supporting its known effect on both OCD-like behavior and hippocampal neuronal differentiation [3,4,42].

A similar approach was used to characterize the possible antidepressant-induced effects on the meninges. We first analyzed the mouse brain retrosplenial meningeal cell number and proliferation following 4 weeks of fluoxetine treatment. Similar to the EE exposure observations, the meningeal nuclei number as well as the number of proliferating cells did not change following the treatment (Figure 5H,I,N,O). We found that while the GLAST^+^ neural precursors showed a trend of increasing, the β3-Tubulin^+^ cells increased significantly in the treated group compared with the control (Figure 5J,K,P,Q) (number of GLAST^+^ cells per 2 mm of cross-sectioned meninges: CTRL = 9.667 ± 0.3333, *n* = 3; FLUOX = 12.67 ± 2.848, *n* = 3; *p* = 0.3545; number of β3-Tubulin^+^ cells per 2 mm of cross-sectioned meninges: CTRL = 15.67 ± 2.848, *n* = 3; FLUOX = 31.19 ± 3.641, *n* = 3; *p* = 0.0284). These data suggest that the meningeal niche responded to fluoxetine-increasing immature neurons, as observed following EE exposure.

Consistently, the number of fractones in meninges was significantly higher in the fluoxetine-treated group when compared with the controls (Figure 5L,R) (number of fractones per 2 mm of cross-sectioned meninges: CTRL = 58.33 ± 3.844, *n* = 3; FLUOX = 82.67 ± 7.446, *n* = 3; *p*= 0.0440), Finally, we assessed the number of macrophages in the meningeal niche following fluoxetine treatment. We observed an increasing trend of CD68^+^ cells in meninges of fluoxetine-treated mice, although the difference was not statistically significant (Figure 5M,S).

Collectively, we found that the meningeal niche reacted to fluoxetine treatment by increasing the immature neurons and the trophic ECM fractones in line with EE meningeal induction. Overall, these results suggest that the meningeal niche may respond not only to EE but also to other pharmacological neurogenic stimuli. Further studies will be necessary to clarify the role of TrkB signaling in the meningeal response to pharmacological neurogenic stimuli.

## 3. Discussion

In this study, we described, for the first time, the meningeal niche response to neurogenic stimuli. EE exposure induced meningeal niche remodeling by modulation of the neural precursor cells, immature neurons, fractones and macrophages. In addition, administration of the TrkB blocker ANA-12 [37] inhibited this response, suggesting that the effects of the EE on the meningeal niche was, at least in part, mediated by BDNF receptor signaling.

Hippocampal neurogenesis has been extensively investigated with pro-neurogenic paradigms like EE and antidepressant treatments. However, the effect of neurogenic stimuli on novel less-known NSC niches, like meninges, is still unexplored [4,10,28]. We first verified the effectiveness of the pro-neurogenic stimuli active in hippocampal neurogenesis, and then we investigated how the meningeal niche reacts to these neurogenic stimuli. Strikingly, EE exposure increased the GLAST^+^ NPs and β3-Tubulin^+^ immature neurons in the meninges without cellular proliferation. The meningeal response to the EE partially differed from the hippocampal one [4,6,28,45,46], as there was no apparent increase in cellular proliferation. However, an overall increase in immature neurons was observed in both the meninges and the hippocampus.

In line with these data, the meningeal niche was already shown to be able to sense and respond to different types of stimuli both in physiological and pathological conditions. Administration of FGF-2 and NGF in the meninges induced hyperplastic changes within the meninges of the rat and monkey [23,24]. Injuries, including spinal cord injuries (SCIs) [25], progressive ataxia [47] and brain stroke [48], were able to increase the number of meningeal-derived doublecortin (DCX)-positive immature neurons.

Aside from the effects on immature neurons and NPs, the pro-neurogenic stimuli are also able to remodel in a significant way the extracellular matrix, as previously described in the brain cortex [49]. Interestingly, we observed an increase in fractones [30], specialized ECM components of the neurogenic niche able to retain trophic factors [50], suggesting an overall remodeling of the meningeal niche.

While the mechanisms underlying the pro-neurogenic effects of neurogenic stimuli have been not entirely clarified, the pivotal role played by BDNF in these contexts has been shown [4,10,13]. We examined BDNF’s role using ANA-12, a small molecule acting as a TrkB non-competitive inhibitor [37]. We observed that the effects on β3-Tubulin and TrkB expression induced by the EE in meninges were partially reverted by ANA-12 administration. The number of CD68^+^ cells was not altered by the TrkB inhibitor, while a statistical reduction in the number of meningeal fractones was observed, suggesting that macrophage activity to generate ECMs may be partially reduced [50].

To identify the origin of the immature neurons emerging in meninges after exposure to the neurogenic stimuli, we took advantage of a transgenic model used for radial glia (RG) cell tracing [32,33]. We observed that the GFP^+^ cells in meninges increased after EE exposure. However, since there was no change in the retrosplenial cortical meningeal cell proliferation, the total increase in GFP^+^ cells may have arisen from migrating progenitors coming from other brain regions. We also detected an increase in the radial glia-derived neural precursors co-expressing TrkB and β3-Tubulin in the meninges following EE exposure, suggesting the RG origin of a part of those populations. The RG origin of neurons present in the meningeal niche was already characterized in a healthy newborn mouse [20]. Here, we showed for the first time their presence in an adult mouse brain. The increase in the number of immature neurons observed in the meninges after the pro-neurogenic stimulus may have different explanations, including (1) differentiation of the RG cells already present in the meninges, (2) other meningeal neural progenitor populations not investigated in this study (including Nestin^+^ and PDGFrβ^+^ cells [20]) or (3) neural precursors migrating from other brain neurogenic niches to the meninges.

We further assessed the meningeal response to fluoxetine, an antidepressant acting as a neurogenic stimulus of the DG [4,5,6]. Consistently, following fluoxetine administration, we observed meningeal niche remodeling through increasing the immature neurons and the ECM fractones. Therefore, similar to the hippocampal niche, the meningeal niche response may not be restricted to the EE, and it may also be generated by other neurogenic stimuli. Previous studies have shown the role of TrkB in anxiety [51] and fluoxetine treatment [13]. However, further studies will be necessary to elucidate the molecular mechanisms driving the meningeal response to pharmacological neurogenic stimuli, including antidepressant treatment.

While the findings regarding the generation of immature neurons in brain meninges after exposure to neurogenic stimuli are completely novel, one question out of many remains to be answered: what is the function of these cells? In recent years, the presence of “standby” neuroblasts expressing markers of neural precursors (like Nestin or GLAST) or of migrating cells (like DCX) while being in a quiescent state (no expression of Ki67 and no BrdU incorporation) was described [14,52,53,54]. Those quiescent cells were found in classical NSC niches like the subgranular [55,56] and the subventricular zone [57] but also in newly described niches like the cortex, the striatum and the meninges themselves [14,20,55,58,59,60]. The role covered by those standby neuroblasts is still an object of speculation and hypothesis, as little is known about their function in the adult brain. Our study showed that, at least in the meninges, quiescent precursors are sensitive to pro-neurogenic stimuli. This opens the stage to further investigations that may help clarify the function of immature neurons in the regeneration or repair processes occurring in an adult brain.

## 4. Material and Methods

### 4.1. Animals

Animal housing and all experimental procedures were approved by the Istituto Superiore della Sanita` (I.S.S., National Institute of Health, Roma, Italy) of Italy and the Animal Ethics Committee (C.I.R.S.A.L., Centro Interdipartimentale di Servizio alla Ricerca Sperimentale, Verona, Italy) of the University of Verona, Italy (authorization number: 237/2016-PR; date of approval: 3 March 2016; protocol number: 56DC9.13). Wild-type (WT) CD1 mice and GLAST-GFP transgenic mice [32,33] were used for all the experiments, as reported in Table 1. The WT CD1 mice were purchased from Charles River Laboratory Italia (Calco, Italy), while the GLAST-GFP transgenic mice were obtained by intercrossing GLAST-CreERT2 mice [32] with the CAG-CAT-EGFP reporter line [33].

The animals were kept in a non-reversed light cycle at a temperature between 20 and 24 °C and humidity between 45 and 60%, and food and water were provided ad libitum.

### 4.2. Exposure to an Enriched Environment

Seven-week-old CD1 male mice (*n* = 6) were housed together in a single rat cage for 1 week. A running wheel and nesting material were always present in the cage, while other toys (e.g., stairs, cardboard rolls and marbles) were added alternatively to the cage to preserve the novelty, according to what is shown in Table 2. The animals were sacrificed after 7 days of EE exposure. Seven-week-old CD1 male mice (*n* = 5) single-housed in a normal cage for 1 week were used as the control animals, as previously described in [61]. The animals were sacrificed 7 days after the start of the experiment.

### 4.3. GLAST-GFP Exposure to EE

The GLAST-Cre^ERT2^ mice [32] intercrossed with the CAG-CAT-EGFP reporter line [33] (GLAST-GFP) allowed for labeling by GFP all the GLAST^+^ cells and their progeny following tamoxifen administration, creating the GLAST-GFP strain. Male GLAST-GFP mice from 7 to 10 weeks old were induced using 3 instances of daily tamoxifen (T5648-1G, Sigma-Aldrich, St. Louis, MO, USA) gavage before the start of the EE protocol. Tamoxifen was dissolved into sunflower seed oil at a 30 mg/mL concentration, and the mice received 3.5 mg of tamoxifen per 35 g of body weight. The animals were left alone for 2 days to recover from the handling and then were either subjected to an EE (*n* = 4) or the control treatment (*n* = 3) as previously described. Observing that the gavage procedure stressed the animals in a significant way, we decided to prolong the EE exposure from 1 to 2 weeks in order to give them time to recover. 

### 4.4. Exposure to the EE and TrkB Inhibitor ANA-12

The 7-week-old CD1 male mice (*n* = 12) were injected intraperitoneally with (1) 10 µL/g of TrkB inhibitor ANA-12 [37] dissolved in sunflower seed oil supplemented with 1% DMSO at a 0.1 mg/mL concentration for 3 consecutive days before the start of the experiment and at the third day of the experiment or (2) the vehicle (sunflower seed oil + 1% DMSO). This administration protocol was adapted from Moy et al. (2019) [38].

After the injections, the mice were divided into four experimental groups: EE ANA-12 (animals exposed to the EE and injected with the inhibitor), EE VEH (animals exposed to the EE and injected with the vehicle), NO EE VEH (single-housed animals receiving just vehicle injections) and NO EE ANA-12 (single-housed animals receiving the inhibitor).

### 4.5. Fluoxetine Administration

The 4-week-old CD1 male mice (*n* = 8) were treated orally with fluoxetine (fluoxetine hydrochloride, LRAA9180, Sigma-Aldrich, St. Louis, MO, USA) for 4 consecutive weeks. The drug was dissolved into the water (0.16 mg/mL concentration) contained in the dispenser normally present in the mice cages, and the mice were able to freely access the water containing the drug. As fluoxetine is light-sensitive, a tinfoil sheet was used to cover the water dispenser in order to avoid any kind of light-induced change in the drug. The drug-containing water was changed by the operator two times per week, and the dispenser was weighted to evaluate the average water consumption for every animal. On the basis of this evaluation, on average, the animals took 29 mg/kg/day of fluoxetine via oral administration. The control group consisted of age-matched CD1 male mice (*n* = 4) which normal water was administered to.

All the animals used in the experiment were administered the marble burying test (MBT), while only *n* = 3 per experimental group were used for subsequent immunofluorescence analysis.

### 4.6. Marble Test Administration

A behavioral marble test was performed at the end of the third week of fluoxetine treatment to preliminarily assess the efficacy of the treatment [62]. The animals were individually placed into a new cage containing 15 equally spaced marbles placed over approximately 5 cm of saw dust, and they were allowed to acclimate for 2 min. Then, their behavior was video-recorded for 30 min. The number of buried marbles (criterium of at least three quarters of its surface under the saw dust) was blindly assessed. All the behavioral testing was performed during the light phase between 12:00 and 4:00 p.m.

### 4.7. Tissue Preparation and Immunofluorescence

At the end of the experimental procedures, all animals were anesthetized by intraperitoneal injection of zoletil (50 mg/kg) and xylazine (7 mg/kg). Once the pedal reflex was lost, the animals were sacrificed by intracardiac perfusion of PBS with a 4% paraformaldehyde (PFA) 4% sucrose (pH 7.4) solution. The brains were extracted, fixed in a 4% PFA solution and transferred into 10% and subsequently 30% sucrose solutions. By cryostat cutting, 35-µm thick medio lateral sagittal brain sections were obtained and processed by immunofluorescence as previously described in [40]. Immunostaining on the cryosections was performed after 30 min of incubation in a blocking solution (PBS 1X with 0.25% Triton X-100, 2% BSA). If required by the specific antibody combination, mouse serum (1:100) was added during incubation in the blocking solution. The sections were then incubated with primary antibodies in the blocking solution overnight at 4 °C. After rinsing 6 times for 5 min in the blocking solution, appropriate secondary antibodies were applied for 4 h at room temperature. After the final washing steps in the blocking solution and then in PBS, nuclear staining with 4′,6-Diamidino-2-phenylindole dihydrochloride (DAPI, Molecular Probes-Thermo Fisher Scientific, Waltham MA, USA) or TO-PRO™-3 iodide (TO-PRO-3, Molecular Probes-Thermo Fisher Scientific) was performed, and the slides were mounted using 1,4-Diazabicyclo[2.2.2]octane (DABCO, Sigma-Aldrich). Staining for the nuclear marker of proliferation Ki67 required a different blocking solution (PBS 1X with 0.5% Triton X-100, 2% BSA).

For immunofluorescence staining using GFP and TrkB antibodies, as they are both produced in the same host, we developed the following protocol using a conjugated primary antibody and a non-conjugated primary antibody.

The cryosectioned sagittal sections were obtained as previously described. Immunostaining was performed after 30 min of incubation in a blocking solution (PBS 1X with 0.25% Triton X-100, 2% BSA). The sections were then incubated with the non-conjugated rabbit primary antibody in a blocking solution overnight at 4 °C. After rinsing 6 times for 5 min in the blocking solution, the appropriate secondary antibody was applied for 4 h at room temperature. Following additional rinsing in the blocking solution 6 times for 5 min, the GFP-conjugated antibody was added to the sections, which were incubated overnight at 4 °C. The final washing steps, nuclear staining and the mounting procedure were performed as previously described.

Control experiments with no primary antibodies were performed to ensure the specificity of the fluorescence staining (Figure S2).

### 4.8. Antibodies

The following primary antibodies were used: anti-GLAST (anti-EAAT1; rabbit, 1:200, Abcam, Cambridge, UK AB416), anti-GLAST (guinea pig; 1:200; Frontier Institute, Ishikari, Japan, AB2571717), anti-DCX (goat, 1:200, Santa Cruz, Santa Cruz, CA, USA, SC-8066), anti-DCX (rabbit, 1:400, Cell Signaling, Danvers, MA, USA, 4604S), anti-β3 tubulin (mouse, 1:400, Promega, Madison, WI, USA, G7121), anti-laminin (rabbit, 1:400, Sigma-Aldrich, St. Louis, MO, USA, L9393), anti-laminin-Alexa Fluor 488 (1:500, Invitrogen, Waltham, MA, USA, PA5-22901), anti-CD68 (rat, 1:200, Invitrogen, Waltham, MA, USA, 14-0681-82), anti-TrkB (anti-tyrosin kinase receptor B, 1:200, rabbit, Santa Cruz, Santa Cruz, CA, USA, SC-12), anti-Ki67 (rabbit, 1:200, Abcam, Cambridge, UK AB16667) and anti-GFP-Alexa Fluor 488 (rabbit, 1:500, Invitrogen, Waltham, MA, USA, A21311).

The following secondary antibodies were used: donkey anti-rabbit Alexa Fluor 488 (1:500, Molecular Probes-Thermo Fisher Scientific, Waltham MA, USA), donkey anti-rabbit Alexa Fluor 647 (1:500, Molecular Probes-Thermo Fisher Scientific, Waltham, MA, USA), donkey anti-goat Alexa Fluor 546 (1:500, Life Technologies-Thermo Fisher Scientific), goat anti-mouse CY3 (1:500, Jackson ImmunoResearch, West Grove, PA, USA), donkey anti-rat CY3 (1:500, Jackson ImmunoResearch, West Grove, PA, USA) and donkey anti-guinea pig CY3 (Jackson ImmunoResearch, West Grove, PA, USA). For nuclear staining, TO-PRO™-3 (1:3000, Molecular Probes-Thermo Fisher Scientific, Waltham, MA, USA) and DAPI (1:2000, Molecular Probes-Thermo Fisher Scientific, Waltham, MA, USA) were used.

### 4.9. Immunofluorescence Image Acquisition, Analysis and Quantification

Immunofluorescence imaging of the brain sections was performed using an Eclipse Ti Nikon microscope (Nikon, Tokyo, Japan) and a Zeiss L710 confocal microscope (Carl Zeiss, Munich, Germany). The acquisition parameter settings (pinhole, gain, offset and laser intensity) were kept fixed for each channel in different sessions of observation at the fluorescence and confocal microscopes. For confocal microscopy, single-plane images were acquired to realize all the quantifications.

Quantification of different markers and nuclei was conducted by counting the positive cells above the basal lamina (identified by laminin reactivity) in at least 15 brain slices for each experimental group (*n* ≥ 3 animals analyzed), analyzing at least five slices from each animal.

At least two images representing 200 μm of meningeal tissue were taken from each slice. A total of at least 2 mm of meninges was analyzed for each animal.

Evaluation of the DG area was realized by designing a user-defined region of interest (R.O.I.) using Fiji-Image J software [63]. The R.O.I.s were delineated while taking into consideration the nuclei composing the DG.

### 4.10. Protein Extraction from the Mouse Meninges and Immunoblot Analysis

After the experimental procedures, the mice (NO EE VEH *n* = 3, EE VEH *n* = 3, EE ANA-12 *n* = 2) were anesthetized by intraperitoneal injection of zoletil (50 mg/kg) and xylazine (7 mg/kg) and sacrificed via decapitation. The brains were quickly extracted, and the meninges and hippocampi were harvested using tweezers under an optical microscope and washed with an HBSS solution (sterile water, HBSS 10X, HEPES 0.3 M, 1% Pen/Strep) and then with PBS1X. The proteins were extracted via mechanical homogenization (GentleMACS^TM^ M tubes, Miltenyi Biotec, Bergisch Gladbach, Germany) in an NP-40 buffer (150 mM NaCl, 1.0% NP-40, 50 mM Tris pH 8.0) in the presence of protease and phosphatase inhibitors. The samples were incubated on ice for 30 min and centrifuged at 10,000× *g* for 15 min at 4 °C. The supernatants were collected and concentrated with centrifugal filters (Amicon Ultra 10 KDa, Merck Millipore, Burlington, MA, USA) according to the manufacturer’s instructions. The protein concentration was determined with a bicinchoninic acid (BCA) protein assay kit (Thermo Scientific, Waltham, MA, USA). The aliquots (25 μg each) were run through a 4–15% SDS–polyacrylamide gel electrophoresis (PAGE) and electroblotted onto a PVDF membrane (Trans-Blot Turbo Transfer System, Bio-Rad Laboratories, Hercules, CA, USA). The membranes were then blocked (EveryBlot Blocking Buffer, Bio-Rad Laboratories, Hercules, CA, USA) and probed with the primary antibodies overnight at +4 °C. The following primary antibodies were used: anti-DCX (Cell Signaling, Danvers, MA, USA cat.no. 4604, 1:500), anti TRK-B (Genetex, Irvine, CA, USA, cat.no. GTX133722, 1:500), anti β3-tubulin (Promega, Madison, WI, USA, cat.no. G7121, 1:500) and anti-p75 (Promega, cat.no. G3231, 1:1000). Subsequently, incubation with anti-mouse or anti-rabbit HRP-conjugated secondary antibodies (Promega, Madison, WI, USA) for 2 h at room temperature was performed. Chemiluminescence-based immunostaining (Clarity Western ECL Substrate, Bio-Rad Laboratories, Hercules, CA, USA) was performed. The images were acquired with the Chemidoc MP Imaging System (Bio-Rad Laboratories, Hercules, CA, USA). Quantitative analyses were performed using Image Lab™ software version 6.0.1 for Windows (Bio-Rad Laboratories, Hercules, CA, USA) and normalizing the total protein content of each lane.

### 4.11. RNA Extraction from the Mouse Brain Meninges and Real-Time (rt) PCR Analysis

After the experimental procedures, animals destined for further Rt-PCR analysis (NO EE VEH *n* = 5, EE VEH *n* = 5, EE ANA-12 *n* = 5) were anesthetized by intraperitoneal injection of zoletil (50 mg/kg) and xylazine (7 mg/kg) and sacrificed via decapitation. After collecting the heads, the skin and skulls were removed to access the brains, which were then extracted. The meninges were harvested using tweezers under an optical microscope and collected into an HBSS solution (sterile water, HBSS 10X, HEPES 0.3 M, 1% Pen/Strep). After centrifugation at 300 *g* per 1 min, the HBSS was substituted with PBS1X and centrifuged at 300 *g* per 1 min. The total RNA was extracted from the fresh mouse meningeal tissue (NO EE VEH *n* = 4, EE VEH *n* = 5, EE ANA-12 *n* = 5) using the RNeasy Plus Micro Kit (Qiagen, Hilden, Germany Cat No. 74034) according to the manufacturer’s protocol, and the RNA abundance was evaluated using the NanoDrop™ One/OneC Microvolume UV-Vis Spectrophotometer (ThermoFisher Scientific, Waltham, MA, USA). Reverse transcription was carried out using a Superscript VILO Master Mix (Invitrogen, Thermo-Fisher Scientific, Waltham, MA, USA). The expression levels of the *ntrk2*, BDNF, *Slc1a3* and *TUBB3* (for primer sequences, see Table 3) genes were quantified by Sybr Green-based real-time PCR (7900HT real-time PCR system, Applied Biosystem, Waltham, MA, USA) according to the ΔΔCt method [64] and by using the *Gaphd* reference gene for data normalization.

### 4.12. Statistics

Data are expressed as the mean ± SEM. Statistical differences were calculated by a two-tailed Student’s t-test or ordinary one-way ANOVA test using GraphPadPrism (GraphPad Inc., La Jolla, CA, USA), where *p* ≤ 0.05 was considered statistically significant.

## Figures and Tables

**Table 1:** Table representing the number of animals used for the different experimental protocols. M = male mice; N.A. = not applicable; IF = immunofluorescence; WB = western blot; RT-PCR = real-time PCR.

Experimental Procedure	Mouse Strain	Sex	Age at Time of Sacrifice	IF	WB	Rt-PCR
Enriched Environment	CD1	M	8 weeks	CTRL *n* = 5 EE *n* = 6	N.A.	N.A.
Enriched Environment on Lineage Tracing Model	GLAST-GFP [32,33]	M	9–12 weeks	CTRL *n* = 3 EE *n* = 4	N.A.	N.A.
Enriched Environment with Administration of TrkB Inhibitor ANA-12	CD1	M	8 weeks	NO EE VEH *n* = 3 EE VEH *n* = 3 EE ANA-12 *n* = 3 NO EE ANA-12 *n* = 3	NO EE VEH *n* = 3 EE VEH *n* = 2 EE ANA-12 *n* = 3	NO EE VEH *n* = 4 EE VEH *n* = 5 EE ANA-12 *n* = 5
Fluoxetine Administration	CD1	M	8 weeks	CTRL *n* = 3 FLUOX *n* = 3	N.A.	N.A.

**Table 2:** Scheme of the toys differentially used to perform the environmental enrichment for the 7-week-old CD1 male mice. An X in a square states that the toy (raw) was present in the cage for that specific day (column). If a cell is empty, it means that the toy (row) was not present for that specific day (column).

Toys	Day 1	Day 2	Day 3	Day 4	Day 5	Day 6	Day 7
Running Wheel	X	X	X	X	X	X	X
Nesting Material	X	X	X	X	X	X	X
Carboard Rolls		X			X		X
Marbles			X		X	X	
Stairs				X		X	X

**Table 3:** Table reporting the primer sequences for the RT-PCR analysis. *TUBB3* is the β3-Tubulin gene, *ntrk2* is the TrkB gene, and *Slc1a3* is the GLAST gene.

Gene Name	Forward Sequence	Reverse Sequence
*BDNF*	CACATTACCTTCCTGCATCTGTTG	CTGGTGGAACATTGTGGCTTT
*TUBB3*	ACAATGAGGCCTCCTCTCACA	TCCATCGTTCCAGGTTCCAA
*ntrk2*	CACACACAGGGCTCCTTAAGG	TGGCGCAAAATGCACAGT
*Slc1a3*	CGCGGTGATAATGTGGTATGC	GAGGCCGACAATGACTGTCA
*Gapdh*	GTCCGTCGTGGATCTGA	GATGCCTGCTTCACCACCTT

## Data Availability

The data that support the findings of this study are available from the corresponding author upon request.

## Supplementary Materials

The following are available online at https://www.mdpi.com/article/10.3390/ijms221910657/s1.

## Abbreviations

BDNF	Brain-derived neurotrophic factors
BrdU	Bromodeoxyuridine
CD68	Cluster of differentiation 68
CNS	Central nervous system
CTRL	Control animal
DCX	Doublecortin
DG	Dentate gyrus
ECM	Extracellular matrix
EE	Enriched environment
FGF	Fibroblast growth factor
FGF2	Fibroblast growth factor 2
FLUOX	Animal treated with fluoxetine
GFP	Green fluorescent protein
GLAST	Glutamate and aspartate transporter
MBT	Marble burying test
NGF	Nerve growth factor
NP	Neural precursors
NSC	Neural stem cells
OCD	Obsessive compulsive disorder
RG	Radial glia
SCI	Spinal cord injury
scRNAseq	single-cell RNA sequencing
SSRI	Selective serotonin reuptake inhibitor
TrkB	Tropomyosin receptor kinase B
WT	Wild-type
β3-Tub	Tubulin β3
