# Supplementary material for: Environmental Enrichment Induces Meningeal Niche Remodeling through TrkB-Mediated Signaling
Source: Int J Mol Sci. 2021 Oct 1;22(19):10657. doi: 10.3390/ijms221910657 (PMC8508649; doi:10.3390/ijms221910657)
Supplement: Supplementary file 1 [file ijms-22-10657-s001.zip › ijms-1269670-suppl.pdf]

International Journal of
Molecular Sciences

MDPI

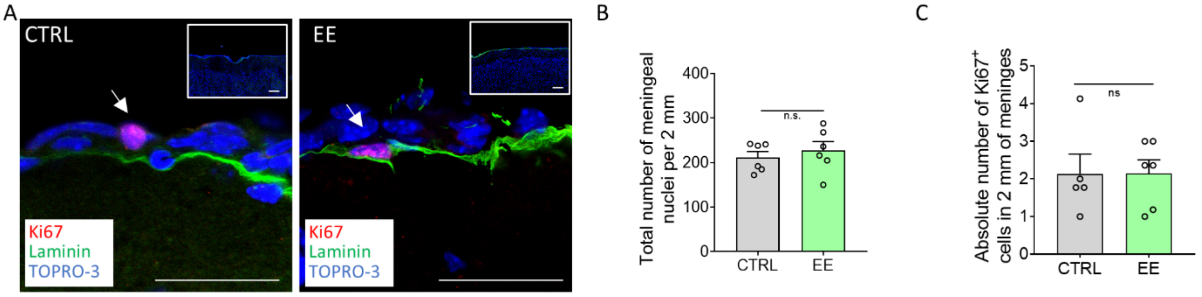

Figure S1 – Enriched environment doesn't induce proliferative changes in meninges (A) Sagittal brain sections of CD1 mice showing Ki67+ (red) cells above meningeal basal lamina (green) in CTRL and EE mice. An insert illustrates a panoramic image where the single-cell level image was taken. (B) Graph showing the number of nuclei in 2mm of retrosplenial brain meninges of CTRL and EE animals. (C) Graph showing the number of Ki67 + cells in 2mm of retrosplenial brain meninges of CTRL and EE animals. Data are presented as mean ± SEM; n.s. = not statistically significant. In picture A nuclei are in blue (TOPRO-3 nuclear staining). Pictures A are maximum intensity projections of z-stack confocal images. Scale bars represent 20µm (A) and 100µm (A, insert). White arrows indicate positive cells.

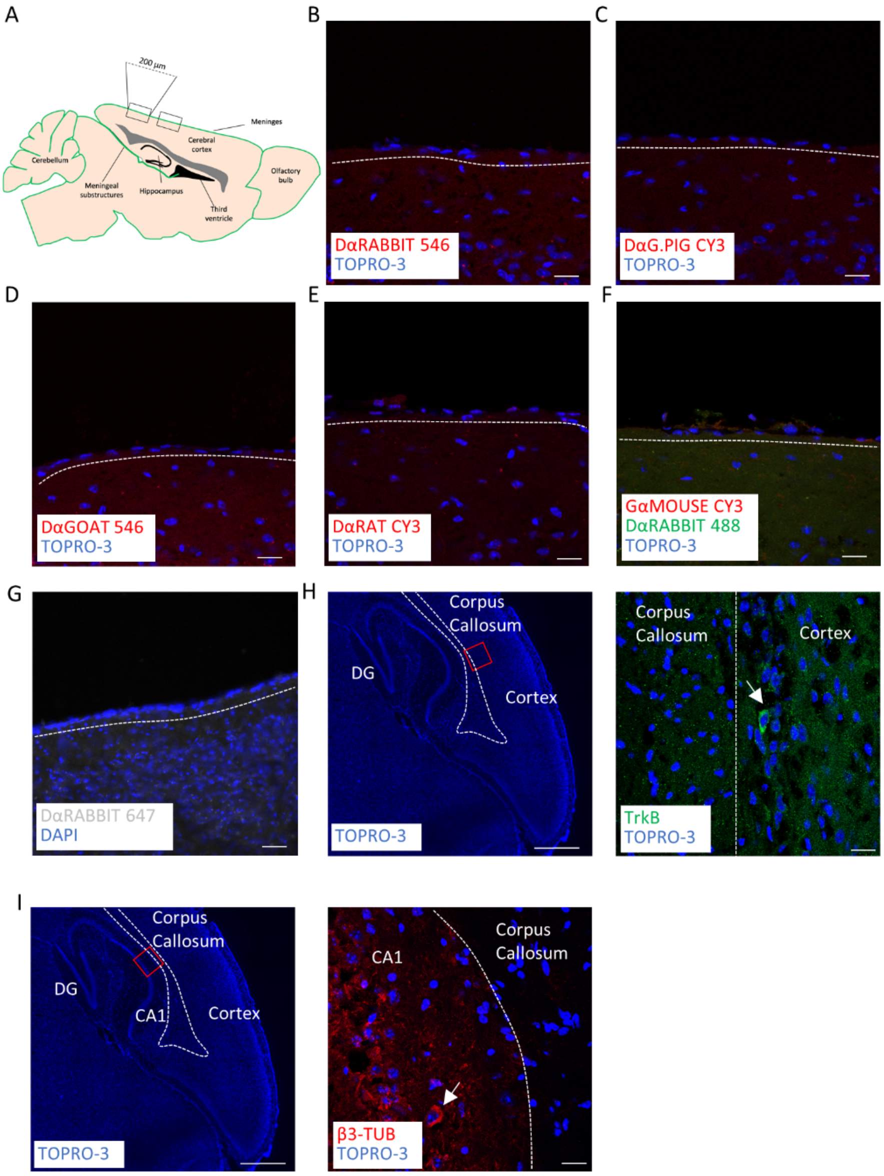

Figure S2 – Immunofluorescence negative controls (A) Scheme depicting the sagittal section of mouse brain highlighting the specific regions where brain external meninges were analyzed. (B-G) Sagittal brain sections of CD1 mice showing no staining when using only the secondary antibodies without any primary antibodies. The secondary antibodies here shown are DonkeyαRabbit Alexa Fluor 546 (B), DonkeyαGuinea Pig CY3 (C), DonkeyαGoat Alexa Fluor 546 (D), DonkeyαRat CY3 (E), GoatαMouse CY3 and DonkeyαRabbit Alexa Fluor 488 (F) and DαRabbit Alexa Fluor 647 (G). (H) Sagittal brain section of CD1 mice showing correct TrkB staining in brain cortex, while showing no staining in Corpus Callosum. (I) Sagittal brain section of CD1 mice showing correct β3-Tubulin staining in hippocampal CA1 area, while showing no staining in Corpus Callosum. All pictures are single plane confocal images. (B-I) Nuclei are shown in blue (TOPRO-3 nuclear staining). Scale bars represent 20μm (B, C, D, E, F, G). In pictures H and I the scale bar for the panoramic image represents 500μm (left), while the scale bar for the magnification is 20μm (right). White arrows indicate positive cells
